# Selenium-Containing Polysaccharide-Protein Complex in Se-Enriched *Ulva fasciata* Induces Mitochondria-Mediated Apoptosis in A549 Human Lung Cancer Cells

**DOI:** 10.3390/md15070215

**Published:** 2017-07-16

**Authors:** Xian Sun, Yu Zhong, Hongtian Luo, Yufeng Yang

**Affiliations:** 1 Institute of Hydrobiology, Jinan University, Jinan 510632, China; imytian@163.com (X.S.); zhongyu@pku.edu.cn (Y.Z.); lhtcoffee@163.com (H.L.); 2 Key Laboratory of Aquatic Eutrophication and Control of Harmful Algal Blooms, Guangdong Higher Education Institutes, Guangzhou 510632, China

**Keywords:** *Ulva fasciata*, selenium-containing polysaccharide-protein complex, apoptosis, mitochondria, reactive oxygen species

## Abstract

The role of selenium (Se) and *Ulva fasciata* as potent cancer chemopreventive and chemotherapeutic agents has been supported by epidemiological, preclinical, and clinical studies. In this study, Se-containing polysaccharide-protein complex (Se-PPC), a novel organoselenium compound, a Se-containing polysaccharide-protein complex in Se-enriched *Ulva fasciata*, is a potent anti-proliferative agent against human lung cancer A549 cells. Se-PPC markedly inhibited the growth of cancer cells via induction of apoptosis which was accompanied by the formation of apoptotic bodies, an increase in the population of apoptotic sub-G1 phase cells, upregulation of p53, and activation of caspase-3 in A549 cells. Further investigation on intracellular mechanisms indicated that cytochrome C was released from mitochondria into cytosol in A549 cells after Se-PPC treatment. Se-PPC induced depletion of mitochondrial membrane potential (Δ*Ψm*) in A549 cells through regulating the expression of anti-apoptotic (Bcl-2, Bcl-XL) and pro-apoptotic (Bax, Bid) proteins, resulting in disruption of the activation of caspase-9. This is the first report to demonstrate the cytotoxic effect of Se-PPC on human cancer cells and to provide a possible mechanism for this activity. Thus, Se-PPC is a promising novel organoselenium compound with potential to treat human cancers.

## 1. Introduction

Due to the increasing incidence of cancer in both developing and developed countries, new chemotherapy compounds are needed [1]. Employing natural or synthetic agents to prevent or suppress the progression of invasive cancers has recently been recognized as an approach with enormous potential [2]. Seaweeds (marine algae) are rich in dietary fiber, minerals, lipids, proteins, omega-3 fatty acids, essential amino acids, polysaccharides, and vitamins A, B, C, and E [3,4,5,6] Studies on the bioactivities of seaweeds reveal numerous health-promoting effects, including anti-oxidative, anti-inflammatory, antimicrobial, and anti-cancer effects. These studies have indicated that marine algae constitute a promising source of novel compounds with potential as human therapeutic agents.

Recently, polysaccharides (PS) from marine organisms have garnered attention because of their potential to be used as ingredients in new medicines and food [7]. PS, including the polysaccharide-protein complex (PPC), are major bioactive constituents of seaweeds with a range of anti-tumor, immune-modulatory, and antioxidant effects [8,9]. However, among marine macrophytes, marine green algae have been less studied than brown and red algae as sources of PPC with such effects. Their antitumor properties have, however, been reported, mainly for those of ulvans. Tabarsa et al. [6] reported that ulvans from *Ulva pertusa* showed little cytotoxicity against tumor cells, but significantly stimulated immunity by inducing nitric oxide and cytokine production.

Selenium (Se) plays an important role in many physiological processes and is therefore an essential trace element for human beings and animals [10] However, organic Se is absorbed more readily and is less toxic than inorganic Se. Se-polysaccharide is reported to be more potent than either Se or polysaccharide. For example, selenylated polysaccharides show greater antioxidant activity than native polysaccharides [11]. Natural plant polysaccharides generally have a low content of Se even in plants grown in high-selenium soil and do not provide adequate dietary Se [12]. Therefore, the use of bioenrichment to prepare high Se polysaccharide is well established and applied by many researchers [13,14]. In our previous study, we found that if *Ulva fasciata* is grown in 500 mg Se/L it can be a source of Se-enriched food because more than 80% of inorganic Se was transformed into the organic form [14].

There is accumulating evidence that bioactive compounds from algae have anticancer effects by inhibition of cancer cell growth, as well as invasion and metastasis. They also induce apoptosis in cancer cells [8]. Apoptosis, programmed cell death, can be induced by both the death receptor and mitochondrial pathways [15]. Apoptotic signals are mediated by Bcl-2 family members, including the anti-apoptotic proteins Bcl-2 and Bcl-xL, and the pro-apoptotic proteins—Bax, Bak, and Bad—in the mitochondrial pathway [16]. The key process of mitochondria-mediated apoptosis is the collapse of mitochondrial membrane potential, which is followed by the translocation of cytochrome c from the mitochondria into the cytosol [17]. Then the subsequent activation of caspases was allowed [18]. The caspase-9 and caspase-3 activated forms are among the main mediators of apoptosis. The two enzymes cleave a wide range of important proteins, including other caspases and the anti-apoptotic protein (such as Bcl-2) [19].

*Ulva fasciata*, also known as sea lettuce, species of the green algal genus *Ulva*, grows abundantly along coastal seashores. Despite the evidence for some biological effect of *Ulva fasciata* against colon cancer cells, there are no available reports of an antitumor effect of Se-PPC from Se-enriched *Ulva fasciata*. Thus, in the present in vitro study, the cytotoxic effect of this Se-PPC on A549 human lung cancer cells was investigated. We aimed to uncover the cytotoxic mechanism of reactive oxygen species (ROS) and mitochondrial apoptosis using various molecular and cellular techniques.

## 2. Materials and Methods

### 2.1. Materials

Sodium selenite (Na_2_SeO_3_), 3-(4,5-dimethylthiazol-2-yl)-2,5-diphenyltetrazolium bromide (MTT), propidium iodide (PI), bicinchoninic acid (BCA) for the protein determination kit, and 2′,7′-dichlorofluorescein diacetate (DCF-DA) were purchased from Sigma (St. Louis, MO, USA). Caspase-3 substrate (Ac-DEVDAMC) was purchased from Biomol (Hamburg, Germany). Caspase-9 substrate (Ac-LEHD-AFC) and caspase-8 substrate (IETD-AFC) were purchased from Calbiochem (San Diego, CA, USA). The primary antibodies used against Cyclin D1, CDK4 p53, Fas, Bax, Bid, Bcl-2, Bcl-XL, and β-actin, were purchased from Santa Cruz Biotechnology (Santa Cruz, TX, USA). The ultrapure water used in all experiments, supplied by a Milli-Q water purification system from Millipore (Billerica, MA, USA).

For the other assays, cells were seeded in 12-well plates at a density of 6 × 10^5^ cells/well.

### 2.2. Preparation, Extraction, and Isolation of Se-PPC

*Ulva fasciata* was collected from the Nanao Island Cultivation Zone (116.6° E, 23.3° N), Shantou, Guangdong, China. Before Se-enriched treatments, the seaweed was acclimated in sterilized seawater for four weeks. Throughout the study, the *Ulva fasciata* was maintained in sterilized seawater enriched by 100 μM of NaNO_3_-N and 10 μM of NaH_2_PO_4_-P at 20 ± 0.5 °C under cool-white fluorescent lamps at 80 μmol photons m^2^ s^−1^. All solutions and glassware were autoclaved at 121 °C for 15 min prior to use.

The seaweed was cultured in 2 L Erlenmeyer flasks containing 1.5 L medium supplemented with Na_2_CO_3_ and sterile air containing 2% CO_2_ as the carbon sources. Se was added in the form of sodium selenite (Na_2_SeO_3_) at concentrations of 500 mg/L. 5 g *Ulva fasciata* FW samples of were placed in each flask which was covered by gauze and placed in indoor tanks at 20 °C, under a light intensity of 275 μmol photons m^2^ s^−1^, at pH 8.0, 30 PSU salinity, and with 12 h:12 h light-dark cycle. Before Se-PPC extraction and isolation, the *Ulva fasciata* thalli were washed three times carefully with ultrapure water to remove the surface Se. 

Ultrasound-assisted extraction (UAE) was performed with a Model VCX-130 ultrasonic processor with a probe horn of 20 kHz frequency and 130 W power (Sonics & Materials Inc., Newton, MA, USA). A 12 mm diameter horn tip was used in the UAE experiments with the power fixed at 70% amplitude (corresponding to an intensity of 26.5 W/cm^2^ tip surface) and the total irradiation period at 60 min (to achieve the maximum Se-PPC yield according to preliminary tests). Each 3 g *Ulva fasciata* sample was mixed with 90 mL of distilled water in a 250 mL plastic centrifuge bottle; the ultrasonic probe was inserted into the sample liquid at ca. 2 cm depth. The sample bottle was immersed in ice with the maximum temperature below 50 °C throughout the UAE period. 

The liquid extract was separated from the solid residues by centrifugation (6000 rpm, 10 min) and subjected to ethanol precipitation (80%, *v*/*v* ethanol) as reported previously [20]. The precipitates were collected after 16,000 rpm, 15 min centrifugation and lyophilized, giving the (crude) Se-PPC fraction. The Se content in Se-PPC was determined by ICP-AES following Sun et al. (2014).

### 2.3. Cell Lines and Cell Culture 

In this study, A549 human lung cancer cells and HK-2 human renal tubular epithelial cells were obtained from American Type Culture Collection (ATCC, Rockville, MD, USA). All cells were cultured in 75 cm^2^ culture flasks in RPMI 1640 (Roswell Park Memorial Institute 1640, Invitrogen, Carlsbad, CA, USA) (for A549 and HK-2) culture medium supplemented with 10% fetal bovine serum (Hyclone, Waltham, MA, USA), 100 units/mL penicillin and 50 units/mL streptomycin in a humidified incubator with an atmosphere of 95% air and 5% CO_2_ at 37 °C. After growth to confluence, the cells were detached with a 0.25% trypsin for passage, and the cells were ready for study until the cell growth was in a stable state and in the logarithmic growth phase unless otherwise specified.

### 2.4. Cell Viability Examination 

The effect of Se-PPC on cell proliferation was determined by the MTT assay. Cells were seeded in 96-well tissue culture plates at 3.0 × 10^3^ cells/well for 24 h. The cells were then incubated with Se-PPC at different concentrations for 72 h. After incubation, 20 μL of MTT solution (5 mg/mL phosphate buffered saline) was added to each well and incubated for 5 h. The medium was aspirated and replaced with 150 mL/well of acidic isopropanol (0.04 N HCl in isopropanol) to dissolve the formazan salt formed. The color intensity of the formazan solution, which reflects the cell growth condition, was measured at 570 nm using a microplate spectrophotometer (SpectroAmaxTM 250, VARIAN, Palo Alto, CA, USA).

### 2.5. Flow Cytometric Analysis

Cell cycle distribution was monitored by flow cytometry. Briefly, cells treated with or without Se-PPC were harvested by centrifugation and washed with PBS. Cells were stained with PI after fixation with 70% ethanol at −20 °C overnight. Labelled cells were washed with PBS and then analyzed by the flow cytometer. The cell cycle distribution was analyzed using MultiCycle software (Phoenix Flow Systems, San Diego, CA, USA). The proportions of cells in G0/G1, S, and G2/M phases were represented in DNA histograms. Apoptotic cells with hypodiploid DNA content were measured by quantifying the sub-G1 peak. For each experiment, 10,000 events per sample were recorded.

### 2.6. Caspase Activity Assay 

Harvested cell pellets were suspended in cell lysis buffer and incubated on ice for 1 h. After centrifugation at 11,000 × *g* for 30 min, supernatants were collected and immediately measured for protein concentration and caspase activity. Briefly, cell lysates were placed in 96-well plates and then specific caspase substrates (Ac-DEVD-AMC for caspase-3, Ac-IETD-AMC for caspase-8, and Ac-LEHD-AMC for caspase-9) were added. Plates were incubated at 37 °C for 1 h. Caspase activity was determined by fluorescence intensity under the excitation and emission wavelengths set at 380 and 440 nm, respectively.

### 2.7. Evaluation of Mitochondrial Membrane Potential (ΔΨm)

Cells in 6-well plates were trypsinized and resuspended in 0.5 mL of PBS buffer containing 10 μg/mL of JC-1. After incubation for 10 min at 37 °C in the incubator, cells were immediately centrifuged to remove the supernatant. Cell pellets were suspended in PBS and then analyzed by flow cytometry. The percentage of the green fluorescence from JC-1 monomers was used to represent the cells that lost Δ*Ψm*.

### 2.8. Western Blot Analysis 

First RIPA lysis buffer (50 mM TriseHCl, 150 mM NaCl, 0.1% SDS, 1% NP-40, 0.5% sodium deoxycholate, 1 mM PMSF, 100 mM leupeptin, and 2 mg/mL aprotinin, pH 8.0) was used to extract total cellular proteins and then the protein extracts were resolved by loading equal amounts of protein, in 10% SDS-PAGEE gel, per lane. They were then put onto Immobilon-P PVDF transfer membranes (Millipore, Bedford, MA, USA) by electroblotting. As a final step, they were blocked with 5% non-fat milk in TBST on a shaker at RT for 1 h.

After that, the membranes were probed by primary antibodies (Cell signaling, Danvers, MA, USA) diluted 1:1000 in 5% nonfat milk at 4 °C overnight, and by secondary antibodies, conjugated with horseradish peroxidase at 1:2000 dilution, at RT for 1 h. To assess the presence of comparable amounts of protein in each lane, the membranes were stripped to detect b-actin (Proteintech group, Chicago, IL, USA). All the protein bands were developed by the SuperSignal West Pico kit (Pierce Biotechnology, Rockford, IL, USA).

### 2.9. Assay for Mitochondrial Cytochrome C Release

This assay was performed according to cytochrome C releasing apoptosis assay kit’s (Biovision, San Francisco, CA, USA) instructions. In brief, after treatment, 1 × 10^6^ cells were pelleted by centrifugation and washed twice with ice-cold PBS. Cell pellets were resuspended with 1 mL cytosol extraction buffer mix containing DTT and protease inhibitor, and incubated for 10 min on ice. After homogenization, unbroken cells and large debris were removed by centrifugation. The resulting supernatants were saved as cytosolic extracts at −70 °C. The pellets were resuspended with 100 mL extraction buffer mix containing DTT and protease inhibitor, and saved as mitochondrial fractions. We loaded 30 mg cytosolic and mitochondrial fractions isolated from A549 cells on 12% SDS-PAGE. Then western blot proceeded with cytochrome C antibody (Biovision, San Francisco, CA, USA).

### 2.10. Measurement of ROS Generation

The effects of Se-PPC on ROS-initiated intracellular oxidation were evaluated by the DCF fluorescence assay. Briefly, cells were harvested, washed with PBS, and suspended in PBS (1 × 10^6^ cells/mL) containing 10 mM of DCFH-DA. After incubation at 37 °C for 30 min, cells were collected and resuspended in PBS. Then, the ROS level was determined by measuring the fluorescence intensity on a Tecan SAFIRE multifunctional monochromator-based microplate reader, with excitation and emission wavelengths of 500 and 529 nm, respectively. Experiments were performed in triplicate.

### 2.11. Statistical Analysis 

Experiments were carried out at least in triplicate and results were expressed as mean ± SD. Statistical analysis was performed using the SPSS statistical package (SPSS 13.0 for Windows; SPSS, Inc., Chicago, IL, USA). The difference between two groups was analyzed by the two-tailed Student’s *t*-test, and between three or more groups by one-way ANOVA multiple comparisons. A difference with *p* < 0.05 (*) or *p* < 0.01 (**) was considered statistically significant.

## 3. Results and Discussion

### 3.1. Cytotoxic Effects of Se-PPC on Various Human Cancer and Normal Cell Lines

**Figure 1:**
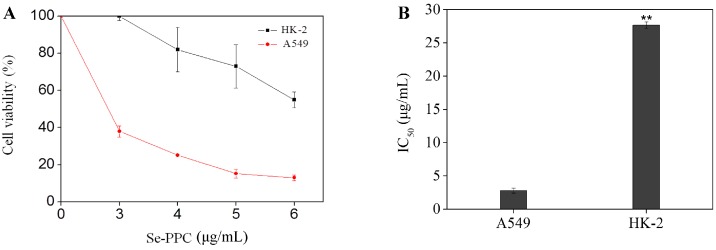
(**A**) Cytotoxic effects of Selenium-containing polysaccharide-protein complex (Se-PPC) on A549 human lung cancer cells and normal cells (HK-2 human renal tubular epithelial cells). Data are expressed as the decrease in cell viability (for MTT assay); (**B**) Growth inhibition of Se-PPC was expressed as the IC_50_ (for MTT assay). Each IC_50_ value represents the mean ± SD of three independent experiments. Cells were treated with Se-PPC for 72 h. All values were obtained at least from three independent experiments. Difference between normal cells and cancer cells with *p* < 0.01 (**) was considered statistically significant.

Many organic selenocompounds have been reported to have potent chemopreventive activities [21]. However, the balance between the therapeutic potential and the toxic effect of a compound is very important when evaluating its pharmacological usefulness. In this study, in vitro cytotoxicities of Se-PPC to A549 cells and normal cells were compared. Se-PPC, from the Se-enriched green seaweed, *Ulva fasciata*, contained 44.4 μg/g Se. The anti-proliferative activities of Se-PPC were first screened against human lung cancer A549 cells in a dose-dependent manner by MTT assay (Figure 1). After the 72 h treatment with Se-PPC at doses of 3, 4, 5 and 6 μg/mL, the percentage of surviving A549 cells were significantly reduced to 37.99, 25.09, 15.26, and 13.05% of the control, respectively (Figure 1A). Exposure for 72 h to 3 μg Se-PPC /mL induced 62.01% A549 (Figure 1A). Se-PPC exhibited broad inhibition on A549 cancer cells with the IC_50_ values of 2.8 μg/mL (Figure 1B). Despite this potency, Se-PPC showed low cytotoxicity toward normal cells (HK-2 renal tubular epithelial cells) with an IC_50_ value of 27.7 μg/mL. These results suggest that Se-PPC selects between cancer and normal cells and has, therefore, potential application in cancer chemoprevention and chemotherapy.

### 3.2. Apoptosis-Inducing Activities of Se-PPC and the Underlying Mechanisms

**Figure 2:**
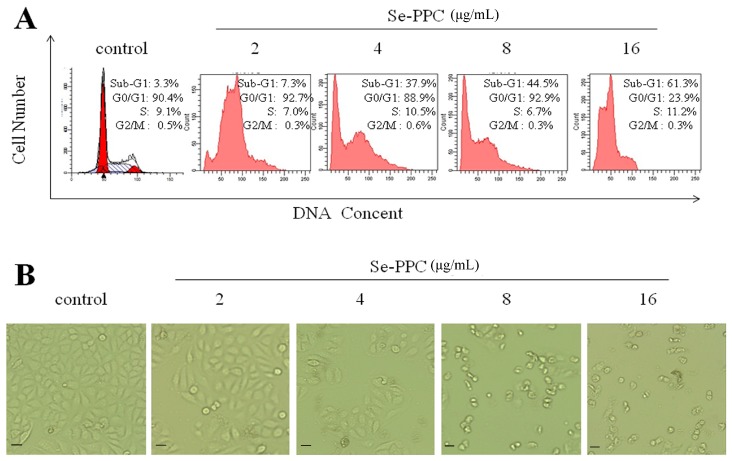
(**A**) Effects of Se-PPC on cell apoptosis and cell cycle distribution in A549 cells (scale bar: 50 μm). The cells treated with different concentrations of Se-PPC for 72 h were collected and stained with PI after fixation, and then analyzed by flow cytometry. Cellular DNA histograms were analyzed by the MultiCycle software. Each value represents the mean of three independent experiments; (**B**) Morphological changes of A549 cells treated with Se-PPC for 72 h observed by phase-contrast microscopy (magnification, 100×). The images shown here are representative of three independent experiments with similar results.

**Figure 3:**
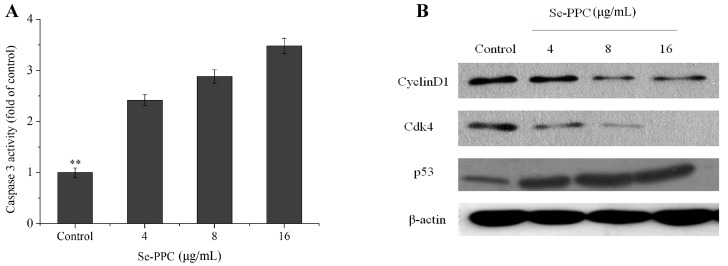
(**A**) Effect of Se-PPC on caspase-3 activity of A549 cells; (**B**) Effect of Se-PPC on cyclin-dependent kinase 4, Cyclin D1, and p53 protein expression of A549 cells. The values represent means ± SD of triplicate determinations. Difference between treatment and control cells with *p* < 0.01 (**) was considered statistically significant.

The inhibition of proliferation of cells treated by Se-PPC could be either the induction of apoptosis or cell cycle arrest, or a combination of the two. The role of apoptosis in the action of anticancer drugs has become clearer [22]. Though, we investigated the underlying mechanism of Se-PPC-induced cell death. El-Bayoumy & Sinha. [21] reported that apoptosis could be critical for cancer chemoprevention by selenocompounds. Our flow cytometry revealed that exposure of the A549 cells to different concentrations of Se-PPC results in a dose-dependent increase in the proportion of apoptotic cells as reflected by the Sub-G1 populations (7.3–61.3%) with a treatment of 2–16 μg/mL Se-PPC (Figure 2A). Moreover, no significant changes in G0/G1, S, and G2/M phases were observed in treated cells. To investigate the potential mechanisms of Se-PPC-mediated induction of cell cycle arrest, the effects of Se-PPC on the expression of CDK4 and Cyclin D1, which are necessary for cell cycle progression, were evaluated. A549 cells were treated with various concentrations of Se-PPC (2–16 μg/mL), and the expression levels of Cyclin D1 and CDK4 proteins were analyzed by Western blot analysis. Se-PPC significantly decreases the protein levels of CDK4 and Cyclin D1 in A549 cells (Figure 3B). In addition, relative to untreated control, Se-PPC suppressed the levels of Cyclin D1 and CDK4 in ovarian cancer cells in a dose-dependent manner (*p* < 0.05). In mammalian cells, cell cycle progression is tightly regulated through the activation of CDKs whose association with the corresponding regulatory cyclins is required for their activation [23]. It is well known that G1 to S phase transition is regulated by complexes formed by Cyclin D and CDK4 [24]. These results indicate that cell death induced by the Se-PPC is mainly due to the induction of apoptosis caused by cell cycle arrest. 

Phase-contrast observations showed that A549 cells treated with Se-PPC exhibited a dose-dependent reduction in cell numbers, a loss of cell-to-cell contact, cell shrinkage, and formation of apoptotic bodies (Figure 2B). Also, the density of cells decreased with the 2–16 μg/mL Se-PPC treatment. Furthermore, when the cancer cells were treated with high concentrations of Se-PPC (16 μg/mL), most of the cells coalesced and were suspended in the culture medium. Se-PPC induced a change in cell morphology and inhibited cancer cell growth in a dose-dependent manner.

The release of cytochrome c from the mitochondria to the cytosol is one of the early events that subsequently lead to apoptosis by activation of caspases, including caspase-3 [25]. The release of cytochrome c into cytosol leads to activation of procaspase-9 in the apoptosome and then causes the cleavage of caspase-3 [26]. Caspase-3, believed to be an important effector protease, is cleaved and activated during apoptosis [25]. For these reasons, we examined the effector caspase (caspase-3) by spectrophotometry; western blot analysis was performed to detect the effect of Se-PPC on the p53 in A549 cells. Our results showed that caspase-3 activities (2.42–3.48 folds) increased significantly (*p* < 0.05) compared to the control, and Se-PPC upregulated the expression of p53 (Figure 3). In cell models, DNA damage activates ATM (ataxia telangiectasia mutated) and ATR (ataxia telangiectasia and Rad3 related) proteins, which signal downstream to checkpoint kinases, such as CHK1 and CHK2. Also, the tumor suppressor gene, p53, which is a major player in the apoptosis because it induces transcription of pro-apoptotic factors and inhibition of pro-survival factors [27]. These results suggest that caspase-3 and p53 contribute to cell apoptosis induced by Se-PPC.

### 3.3. Mitochondria Plays an Important Role in Se-PPC-Induced Apoptosis

**Figure 4:**
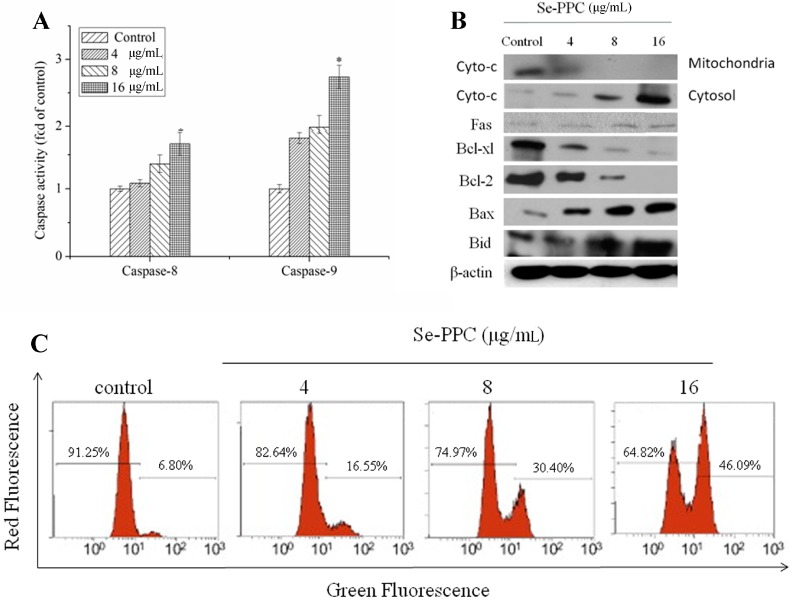
(**A**) Effect of Se-PPC on caspase-8 and caspase-9 activities of A549 cells; (**B**) Effect of Se-PPC on cytochrome C, Fas, Bcl-2, Bcl-XL, Bid, and Bax protein expression of A549 cells; (**C**) Cells treated with Se-PPC were harvested and stained with the mitochondria-selective dye JC-1 and then analyzed by flow cytometry. The number in the right region of each dot plot represents the percentage of cells that emit green fluorescence due to the depletion of Δ*Ψm*. The values represent means ± SD of triplicate determinations. Difference between treatment and control cells with *p* < 0.05 (*) was considered statistically significant.

Generally, apoptosis occurs via death receptor-dependent (extrinsic) or mitochondrial (intrinsic) pathways. The mitochondrial pathway of cell death is mediated by Bcl-2 family proteins which disrupt the mitochondria membrane potential and result in release of apoptogenic factors such as cytochrome c, Smac/Diablo, and AIF, into the cytosol [28]. Cytochrome c then forms an apoptosome containing apoptosis activating factor 1 and caspase-9, which then activates the downstream apoptotic signals [22]. In this content, we examined the cytochrome c levels in cytosol fractions of cells treated with Se-PPC at doses of 4, 8, and 16 μg/mL. The result of western blot showed that the cytosolic cytochrome c protein expression increased markedly in a dose-dependent manner in Se-PPC-treated cells (Figure 4B). Since release of cytochrome c into the cytosol is usually preceded or accompanied by a loss or disruption of mitochondrial membrane potential and this collapse is an essential step occurring in cells undergoing apoptosis [29]. Loss of Δ*Ψm* is associated with the activation of caspases and the initiation of apoptotic cascades. Thus, the status of mitochondria in A549 cells exposed to Se-PPC was investigated by JC-1 flow cytometric analysis. It was found that Se-PPC induced significant depletion of Δ*Ψm* in A549 cells (Figure 4C). The percentage of cells with depolarized mitochondria increased from 6.80% (control) to 16.55% (4 μg/mL), 30.40% (8 μg/mL), and 46.09% (16 μg/mL), respectively. 

Caspases are a family of cysteine proteases that play central roles in the initiation and execution of apoptosis [21] The extrinsic pathway is triggered by activation of death receptors. The formation of a death-inducing signaling complex subsequently activates initiator caspase-8 [30]. Because caspases have been identified as targets for therapeutic intervention using fluorimetry, we measured the activity of two initiator caspases, caspase-8 (Fas/TNF-mediated) and caspase-9 (mitochondrial-mediated). Our results showed that Se-PPC-evoked apoptosis resulted in dose-dependent activation of caspase-8 and caspase-9 in A549 cells, suggesting that both caspase-8 and caspase-9 were involved in Se-PPC-induced apoptosis (Figure 4A). Activity of initiator caspase-9 increased 1.75–2.74-fold in cells exposed to 4–16 mM of Se-PPC compared with controls. In contrast, little increase in activities of caspases-8 (1.08–1.66-fold) in response to Se-PPC treatment was observed. Meanwhile, under Se-PPC treatments, the expression of Fas in A549 cells were upregulated insignificantly (Figure 4B), suggesting that the contribution of caspase-8 to the induction of cell apoptosis was likely to be insignificant, and that the mitochondrial-mediated apoptotic pathway played the major role in Se-PPC-induced apoptosis in A549 cells. Because it was well-known that caspase-8 is activated via the death receptor-mediated pathways.

The Bcl-2 family was divided into two major categories, namely anti-apoptotic proteins (Bcl-2 and Bcl-XL) and pro-apoptotic proteins (Bax and Bid) [31]. Pro-survival family members associate with the mitochondrial outer membrane and maintain their integrity. In contrast, pro-apoptotic members such as Bax and Bid oligomerize in the outer membranes of the mitochondria and disrupt their integrity, causing the release of apoptogenic factors [28]. In this study, under Se-PPC treatments, the expression of Bax and Bid in A549 cells were upregulated, but the expression of Bcl-2 and Bcl-XL were downregulated (Figure 4B). These results indicated that Se-PPC induces mitochondria-mediated apoptosis by regulating the expression of Bcl-2 family proteins.

### 3.4. Oxidative Stress Is Involved in Se-PPC-Induced Apoptosis

**Figure 5:**
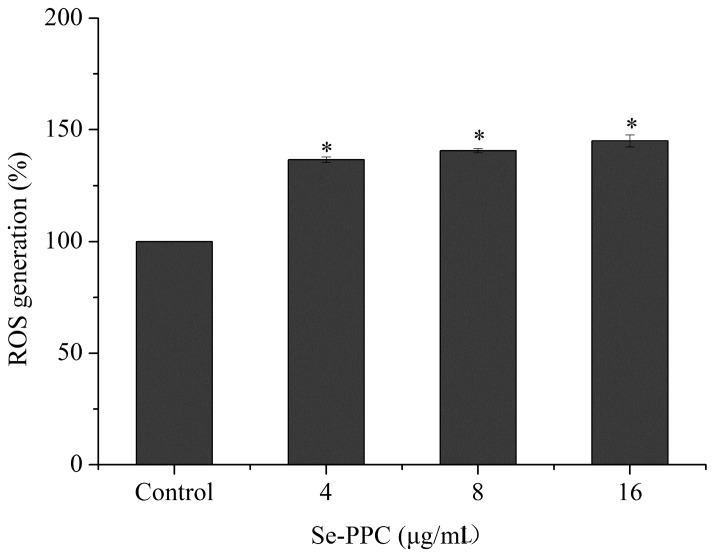
ROS overproduction in A549 cells induced by Se-PPC as determined by DCF fluorescence assay. Cells were treated with indicated concentrations of Se-PPC for 24 h. All experiments were carried out at least in triplicate. Difference between treatments and control with *p* < 0.05 (*) was considered statistically significant.

Many chemopreventive and chemotherapeutic agents have been found to induce cancer cell apoptosis through upregulation of intracellular reactive oxygen species (ROS) generation [32]. Letavayova, Vlckova & Brozmanova [33] suggested that the toxicity of Se is due to the induction of oxidative stress and disruption of redox homeostasis. The mitochondrial respiratory chain is a potential source of ROS [34]. ROS—including the superoxide anion, hydrogen peroxide, and hydroxyl radical—are produced under normal aerobic growth conditions within cells, but they are elevated under the influence of external stimuli. Intracellular ROS may attack cellular membrane lipids, proteins, and DNA and also cause oxidative injury [35]. Considerable evidence has suggested that DNA damage can cause cell death by induction of apoptosis via various signaling pathways [27]. Some selenocompounds have been reported to have the potential to induce DNA damage [33]. Nilsonne et al. [36] have provided evidence that ROS generation acts as an important cellular event induced by Se compounds and results in cell apoptosis and/or cell cycle arrest. The involvement of oxidative stress in Se-PPC-induced apoptotic cancer cells was investigated to gain insight into the mechanism of the cytotoxic action of Se-PPC. Our results showed that treatments with Se-PPC generated a dose-dependent increase in DCF fluorescence intensity, indicating upregulation of intracellular ROS levels, suggesting that ROS is a critical mediator in Se-PPC-induced cell apoptosis in A549 cells (Figure 5).

## 4. Conclusions

In conclusion, it is shown for the first time that Se-PPC is a novel anti-proliferative agent with a broad spectrum of inhibitions against A549 cancer cells via the induction of apoptosis. However, Se-PPC was found to show a low cytotoxicity toward HK-2 renal tubular epithelial cells. Overproduction of ROS contributes to Se-PPC-induced apoptosis, which in turn leads to apoptotic signals including mitochondria- and caspase-dependent processes in human lung cancer A549 cells. These findings indicate that Se-PPC is a promising organoselenium agent for the treatment of human cancers.
